# Open-label add-on treatment trial of minocycline in fragile X syndrome

**DOI:** 10.1186/1471-2377-10-91

**Published:** 2010-10-11

**Authors:** Carlo Paribello, Leeping Tao, Anthony Folino, Elizabeth Berry-Kravis, Michael Tranfaglia, Iryna M Ethell, Douglas W Ethell

**Affiliations:** 1 Surrey Place Centre, Toronto, Ontario, Canada; 2 Departments of Pediatrics, Neurological Sciences, Biochemistry, Rush University Medical Center, Chicago, IL, USA; 3 FRAXA Research Foundation, Newburyport, MA, USA; 4 Biomedical Sciences, University of California, Riverside, CA, USA; 5 Biomedical Sciences, Western University of Health Sciences, Pomona, CA, USA

## Abstract

**Background:**

Fragile X syndrome (FXS) is a disorder characterized by a variety of disabilities, including cognitive deficits, attention-deficit/hyperactivity disorder, autism, and other socio-emotional problems. It is hypothesized that the absence of the fragile X mental retardation protein (FMRP) leads to higher levels of matrix metallo-proteinase-9 activity (MMP-9) in the brain. Minocycline inhibits MMP-9 activity, and alleviates behavioural and synapse abnormalities in *fmr1 *knockout mice, an established model for FXS. This open-label add-on pilot trial was conducted to evaluate safety and efficacy of minocycline in treating behavioural abnormalities that occur in humans with FXS.

**Methods:**

Twenty individuals with FXS, ages 13-32, were randomly assigned to receive 100 mg or 200 mg of minocycline daily. Behavioural evaluations were made prior to treatment (baseline) and again 8 weeks after daily minocycline treatment. The primary outcome measure was the Aberrant Behaviour Checklist-Community Edition (ABC-C) Irritability Subscale, and the secondary outcome measures were the other ABC-C subscales, clinical global improvement scale (CGI), and the visual analog scale for behaviour (VAS). Side effects were assessed using an adverse events checklist, a complete blood count (CBC), hepatic and renal function tests, and antinuclear antibody screen (ANA), done at baseline and at 8 weeks.

**Results:**

The ABC-C Irritability Subscale scores showed significant improvement (p < 0.001), as did the VAS (p = 0.003) and the CGI (p < 0.001). The only significant treatment-related side effects were minor diarrhea (n = 3) and seroconversion to a positive ANA (n = 2).

**Conclusions:**

Results from this study demonstrate that minocycline provides significant functional benefits to FXS patients and that it is well-tolerated. These findings are consistent with the *fmr1 *knockout mouse model results, suggesting that minocycline modifies underlying neural defects that account for behavioural abnormalities. A placebo-controlled trial of minocycline in FXS is warranted.

**Trial registration:**

ClinicalTrials.gov Open-Label Trial NCT00858689.

## Background

Fragile X Syndrome (FXS) is the most common known inherited form of intellectual disability and autism, with an estimated prevalence of about 1/4000 males and females [1]. It is also associated with a range of learning disabilities, neurological problems such as seizures [2], and behavioural difficulties. For many individuals with FXS, their behavioural difficulties result in severe problems within the family and community, particularly in the form of agitation, temper outbursts, hyperactivity, and aggression [3,4].

In 1991 the gene responsible for FXS was identified and named *Fragile X Mental Retardation-1 (FMR1) *[5]*FMR1 *is located toward the end of the long arm of the X chromosome at Xq27.3. Most FXS cases are the result of an unstable trinucleotide repeat (CGG) expansion in the 5'-untranslated region of *FMR1*. When of sufficient size, these expansions cause promoter methylation and gene silencing, resulting in the absence of, or reduced levels of *FMR1 *mRNA and FMRP (the protein product of *FMR1*). FMRP is an RNA binding protein that associates with actively translating ribosomes in dendrites [6,7]. That discovery led to the hypothesis that FMRP regulates synapse-relevant protein synthesis, which may affect synapses in an activity-dependent manner [8,9]. FMRP deficiency would therefore alter synaptic plasticity and account for the cognitive and behavioural impairments associated with FXS [10,11]. Measures of cognition, including IQ, correlate with lymphocyte levels of FMRP in blood in both males and females affected by FXS [12-14]. Functional MRI (fMRI) studies have shown that lymphocyte levels of FMRP also correlate with brain metabolism during tasks involving math calculations, working memory and response inhibition [15-17].

It has been hypothesized that the absence of FMRP disrupts regulation of group 1 metabotropic glutamate receptor (mGluR and mGluR5)-dependent translation in dendrites [10]. The defects in dendritic spine maturation that have been found in the brains of patients with Fragile X suggest that these structures may represent an anatomical and physiological basis for the cognitive deficits associated with this disorder [18,19].

The treatment of patients with Fragile X syndrome often requires a variety of psychopharmacological and behavioural approaches [20-22]. Although a variety of medications can be helpful in FXS, only lithium has been studied as a potential targeted intervention based on molecular abnormalities seen in the FX drosophila and mouse models [23]. Lithium was found to reduce mGluR-activated translation and reversed phenotypes in the *dfxr *mutant fly and *fmr1 *knockout mouse.

Investigation into the developmental effects of matrix metalloproteinases (MMPs) on hippocampal neurons has shown that MMPs can influence dendritic spine development and hence synaptic stability [24]. Recent findings have suggested that the MMP-inhibiting activity of minocycline may provide specific benefits in the treatment of FXS. Minocycline has been found to inhibit the activity of matrix metallo-proteinase-9 (MMP-9), which is elevated in the hippocampus of *fmr1 *KO mice and may be partially responsible for the immature dendritic spine profile of hippocampal neurons [25]. Minocycline treatment of *fmr1 *KO mice rescued the spine phenotype in *fmr1 *KO hippocampal neurons, both in vitro and in vivo. Minocycline treated *fmr1 *KO mice also performed significantly better in the elevated plus-maze, a cognitive performance test that measures activity and anxiety.

These new findings in the mouse model of FXS and our new understanding of the neurobiology of FXS have suggested that the benefits of minocycline treatment should be evaluated across a broad range of cognitive and behavioural measures in adolescents and adults with FXS. This pilot study was initiated to test the concept that minocycline is a specific molecular targeted treatment for FXS that will display beneficial effects on disruptive behaviour and possibly other associated features of FXS via a reduction in MMP-9 activity.

## Methods

### Research Design and Methods

The overall design for this study was an open-label, add-on pilot trial of minocycline in participants with FXS, including adolescents and young adults. Twenty subjects with FXS were enrolled and after baseline testing, they were started on an 8-week treatment course of minocycline added on to any other medications being administered at the time of enrollment. Trial length for this acute treatment period was chosen for several reasons. Firstly, the time course of FXS response to minocycline is not certain and some studies which measured aggression while using a treatment duration of less than 8 weeks, were unable to detect drug-placebo differences that did become apparent in trials of longer duration. Furthermore, shorter trial durations tend to be more vulnerable to positive expectation bias, as seen for example in the clinical trial using amantadine to treat autism [26].

### Subjects

It was anticipated that minocycline may be beneficial for affected individuals of all ages and this trial included both males and females affected with FXS between 13 and 35 years of age. Adolescents and young adults were included because this is an age group that often presents with impairing problems, with aggression, agitation, and mood instability. The use of minocycline during tooth development (last half of pregnancy, infancy and childhood under the age of 13 years) may cause permanent tooth discolouration (yellow-grey-brown), so this trial did not include patients under the age of 13.

Subjects were recruited from the FXS Clinic at Surrey Place Centre or were self-referred after learning about the study from the Fragile X Research Foundation of Canada website or Newsletter. Individuals enrolled must have met at least a Clinical Global Impressions-Severity Scale (CGI-S) rating of moderate impairment (score of 4 or higher) so that the risk of exposing them to a medication was justified by a moderate level of behaviour difficulty. No changes in psychoactive medications were allowed during the 8-week treatment period. Each subject was required to be in good physical health as determined by the screening procedures described below.

Inclusion criteria included (1) diagnosis of FXS by clinical evaluation and confirmed by *FMR1*-DNA testing with presence of full mutation or mosaicism for the full mutation. Prior DNA test reports were accepted, when available (2) age between 13 to 35 years inclusive at the time of informed consent, (3) male or female, (4) CGI-Severity Score of at least 4, indicative of moderate or greater severity of behavioural problems, (5) a score of 9 or more on the Aberrant Behaviour Checklist - Irritability Scale (top 50th %-tile), (6) availability of parent and/or caregiver for all clinic visits and assessments, (7) English language fluency and reading level of 6th grade or greater in one caregiver (8) stable doses of psychoactive medications for at least 8 weeks prior to entry into the study, and (9) for those subjects with a seizure disorder, a stable regimen of anticonvulsants for at least four months prior to entry into the study.

Subjects were excluded from participation if they had (1) a known allergy to minocycline or tetracycline, (2) kidney disease or elevated renal function tests, (3) liver disease or elevated liver function tests, (4) neutropenia, anemia, or thrombocytopenia, (5) history of systemic lupus erythematosus or screening anti-nuclear antibody (ANA) titre of > 1:40, as minocycline may cause a lupus-like reaction, (6) individuals who did not have a parent or caregiver who was willing to participate in the clinic visits, (7) individuals who were pregnant or at risk of becoming pregnant, specifically sexually active females were excluded. (8) Presence of persistent psychotic symptoms (9) subjects with symptom severity judged to likely endanger personal safety or the safety of others or which would preclude co-operation for necessary tests. Informed written consent was obtained from either the subject or the parent prior to participation. Assent from the subject was obtained in every case in which the subject was not his own legal guardian and had sufficient cognitive ability to agree to participate. The study was approved by the Research Ethics Board at Surrey Place Centre.

### Baseline Evaluation

All subjects had a baseline medical evaluation, which included detailed history, medication review, review of FXS test results, a physical exam and screening blood tests, including a CBC, BUN, creatinine, ALT, AST, ANA to assess medical health. Female patients also had a serum Beta HCG level done. The Aberrant Behaviour Checklist (ABC-C) was completed by the caregiver, along with the visual analog scale for behaviour (VAS). The Clinical Global Impression - Severity (CGI-S) scale for severity of behavioural dysfunction was completed by the PI (CP).

### Minocycline Dosing

All subjects were given minocycline capsules to be taken orally on a BID basis to minimize gastric upset. In order to determine the optimum effective dose, all subjects were randomly assigned to either a low-dose group or a high-dose group. Subjects in the low-dose group received 50 mg of minocycline BID. Subjects in the high-dose group were started on 50 mg of minocycline BID but had their dose increased to 100 mg BID at the 2 week follow-up visit. These are standard doses used in general practice and were used in this trial because there is already a well established safety and side-effect profile at these doses. Any subjects who were unable to tolerate the higher dose would have been allowed to continue at the lower dose. However, none of the participants in this study required a dosage adjustment during the 8 week treatment period.

### Safety, Compliance and Adverse Event Monitoring

Initial blood tests for safety monitoring included a complete blood count (CBC), blood urea nitrogen (BUN), creatinine, ALT, aspartate aminotransferase (AST), ANA, and pregnancy testing for women of childbearing potential. The CBC, ALT, AST, BUN, creatinine and ANA levels were measured again in 8 weeks. Outcome measures were conducted at baseline and at 8 weeks. A clinical visit occurred at enrolment, baseline, 2 weeks, 4 weeks, and 8 weeks. Compliance was checked at each clinic visit with a direct query for any missed doses. A medication dose tracking sheet was given to the parent or caregiver to document the date and time of every dose given. At each clinic visit a capsule count was done on each medication bottle. At each visit, subjects and families with less than an average of 90% compliance up to that date were counselled about the importance of taking the minocycline as dosed. Anyone with 80% or less compliance required discussion with the PI about continued study participation.

### Assessment of Adverse Events

Caretakers were questioned regarding health problems, intercurrent illnesses and concomitant medications at each clinic visit. Any adverse event was documented regarding time it occurred, duration, severity and whether it was considered related to the minocycline or not.

At each visit the caregiver was asked to fill out a side effects questionnaire that consisted of 34 items including sedation, energy level, chest pain, nausea, vomiting, bowel and bladder problems, fever, rash, joint pains and swelling, infections and changes in teeth or skin colour, headaches or visual disturbances. These items cover all of the known side effects of minocycline.

### Primary Outcome Measure

#### Aberrant Behaviour Checklist-Community (ABC-C)

Our main outcome measure was the ABC Irritability subtest score because previous experience with patients in this population suggested that it would be the most sensitive to the benefits of minocycline. The ABC Irritability subscale was used as the primary outcome measure and was expected to support the theory that minocycline is a specific molecular targeted treatment for FXS that will display beneficial effects on disruptive behaviour and possibly other associated features of FXS. This theory would be supported by an improvement in the irritability score after 8 weeks of minocycline treatment. The ABC is a global behaviour checklist implemented for the measurement of drug and other treatment effects in cognitively impaired individuals. It is made up of five empirically derived dimensions including irritability, lethargy/withdrawal, inappropriate speech, hyperactivity, and stereotypic behaviour based on 58 items that describe various behavioural problems. The ABC was validated on 509 (moderate to profound) cognitively impaired residents (M = 26 years old, SD = 14.5, ages 6 and up). Spearman correlation coefficients for subscales were very high, from .96 to .99, and test-retest reliability is good [27]. The 15-item Irritability Scale includes questions about aggression, self-injury, tantrums, agitation, and unstable mood on a scale of 0 to 45 with higher scores indicating greater severity. This scale has been successfully used in previous medication studies in children with autism [28] and in patients with FXS and in a controlled trial of ampakine CX516 in FXS [29]. All ABC subscales showed good reliability when used by parents and caregivers of individuals with FXS to assess behaviour in the CX516 study, and yielded intraclass correlation coefficient (ICC) values of 0.7-0.9.

### Secondary Outcome Measures

#### Subscales of the ABC-C

Secondary outcome measures were the other subscales of the ABC-C including the Lethargy (score range 0 - 48), Hyperactivity (score range 0 - 48), Stereotypy (score range 0 -21), and Inappropriate Speech (score range 0 -12) Subscales.

#### Visual Analog Scale (VAS)

This methodology involves a parent-defined target behaviour and has been utilized and validated in the RUPP clinical trials particularly for the risperidone studies in autism [28,30]. The parent or caretaker chooses a behaviour target symptom, such as tantrums or aggression. This allows the problems that are of concern to parents and the family to be targeted in the trial. The use of this methodology was originally described by Arnold et al [31] and subsequently utilized in studies of ADHD symptoms [32,33]. In this study the family chose a target symptom at baseline and then continued with that symptom throughout the eight weeks of the study. The caregiver described the symptom including frequency, duration, time spent on it and intensity, interference in daily function or family life and other consequences. These responses were documented in a written format. We asked the caregiver to mark on a visual analogue scale (VAS), a 10 centimetre line, where the symptom lies from worst ever to no problem at all.

In the RUPP 8 week controlled trial of risperidone in autism the Parent Defined Target Symptom Scale was a valid measure of outcome and correlated (Pearson r) with the Irritability subscale of the ABC at 0.64 and with the CGI-I at 0.75 [26]. The Parent Target Symptom score demonstrated a significant difference between risperidone and placebo at weeks 4 and 8 [28]. The VAS with the Parent Target Symptom score was used for participants with FXS in the CX516 study. The visual analogue scale showed good reliability when used by parents and caregivers of individuals with FXS to assess behaviour in the CX516 study with an ICC value of 0.8.

#### Clinical Global Impressions Scale-Severity (CGI-S) and Improvement (CGI-I)

This scale is commonly used in drug studies because it allows the clinician to utilize the history from the parents or caretaker and incorporate it into a clinical rating first for severity and then for the clinical follow up of the patient. In the initial evaluation of each participant, we used the CGI-S to judge the severity of the symptoms requiring a rating of moderate or higher for inclusion in the study. In the follow up clinical visits we used the CGI-I. This 7- point scale is a well validated measure of clinical global impression of improvement that the clinician fills out after considering all the available new information on the participant including the parent history, the examination in clinic, reports from the school and other sources [34].

### Statistical Analysis

Data were analyzed for change at 8 weeks treatment from baseline for all safety and outcome measures. A series of paired samples t-test were conducted on all outcome measures and mean group change and standard deviations were determined for all measures for which sufficient data existed from both assessments. Analysis involving the ABC-C assessed pre-post differences across the 5 separate subscales and the Total Behaviour Problems. To address the issue of multiple comparisons, Bonferroni correction procedures were used for all analyses involving the ABC-C scores [35]. More specifically, since analysis involving the ABC-C included 5 separate comparisons, a more conservative level of significance was calculated by dividing the original significance value (.05) by the number of simultaneous tests conducted (5), to yield a new significance value of .01. Distributions were within the normal range, as indicated by skewness and kurtosis across variables.

Due to the exploratory nature of the investigation, participants were randomly assigned to a low dose or high dose group to assess if there could be a dose response effect. These results were assessed using a series of repeated measures analysis of variance (ANOVA). For this analysis the independent variables were dose (high dose vs. low dose) and time (baseline vs.8 week follow-up). Consistent with previous analysis, the dependent variables included all subscales of the ABC-C, CGI, and VAS. Also, 10 participants were enrolled in the study while currently taking other types of medication. In order to assess potential drug interaction effects, a series of repeated measures ANOVA were conducted. For this analysis, the independent variables were medication group (Minocycline only group vs. Minocycline plus additional medication(s) group) and time (baseline vs. 8 week follow-up). Consistent with previous analysis the ABC-C, CGI, and VAS served as the dependent measures.

## Results

Twenty subjects were enrolled into the study and started on minocycline. One subject dropped out of the study after four weeks of treatment due to side effects. Nineteen subjects completed the minocycline treatment protocol for the full 8 week treatment period. Demographic characteristics of the study population are shown in Table 1.

**Table 1:** Demographic Data for Subject Cohort in Open-Label Treatment Study of Minocycline in FXS

Age	
Mean +/- SD (range)	18 +/- 5

Range	13 to 32

**Sex**	

Male	18

Female	2

**Race**	

Caucasian	18

Asian	2

**Living Setting**	

Home with family	20

**Number of concomitant psychoactive** **medications**	

None	10

One	4

Two or more	6

**Type of concomitant psychoactive** **medications**	

SSRI's	4

Stimulants	5

Antipsychotic	1

Anticonvulsants	4

### Safety and Adverse Events

There were no clinically significant changes in blood chemistries including liver functions, creatinine, BUN, or blood counts during the eight week treatment period. However, two participants developed an asymptomatic seroconversion of their ANA, both exhibiting a 1/80 titre with a nucleolar pattern.

There was no significant change in heart rate, blood pressure or weight for any study subject. Adverse events observed during the treatment period are shown in Table 2. There were no serious adverse events. Dizziness was reported by 4 subjects, diarrhea by 3 subjects, sleepiness and headache each reported by 2 subjects. The adverse events reported were transient, mild or moderate in intensity, and only one subject withdrew from the trial after four weeks of treatment because of flu-like symptoms that we could not definitely attribute to the use of minocycline. Of the two individuals who had seizures while on minocycline, one had no change in seizure frequency or severity from the period prior to starting minocycline and was therefore considered unrelated to minocycline treatment. The second had three minor petit mal seizures over a three day period, compared to four seizures over the one year period prior to starting the minocycline. It was concluded that the minocycline was an unlikely cause of these seizures.

**Table 2:** Adverse Events During Minocycline Treatment Period

Symptom	**Time Period**^**1**^		**Total Events**^**2**^	**Total Subjects**^**3**^	Relationship to Minocycline Treatment				
	0-4	4+			Unrelated	Unlikely	Possible	Probable	Definite
Diarrhea	3		3	3					✔

Abdominal pain	1	1	2	2	✔				

Nausea	1		1	1			✔		

Fatigue	2		2	2			✔		

Dizziness	2	3	5	4				✔	

Sleepiness		2	2	2				✔	

Headache	1	3	4	2				✔	

Pruritus		2	2	2			✔		

Arthralgia		2	2	2	✔				

Epistaxis	1		1	1	✔				

Odynophagia			1	1	✔				

Dark urine		1	1	1		✔			

Seizure		2	4	2		✔			

Labs									

+ANA		2	2	2					✔

^1 ^Time period in weeks such that 0-4 represents the first 4 weeks of treatment and 4+ represents the time between 4 weeks and 8 weeks.

^2 ^Total events is the total number of events that occurred during the treatment period.

^3 ^Total subjects is the total number of subjects experiencing the event (some subjects had an event more than once).

### Efficacy

There was significant improvement in behaviour across the cohort as measured by 4 out of the 5 subscale scores of the ABC-C during the period of minocycline treatment (Table 3). These included the irritability subscore (primary outcome measure) as well as the stereotypy, hyperactivity and inappropriate speech subscales. The lethargy subscore was the only area that did not show a significant change. The CGI also showed statistically significant improvement with only one subject remaining unchanged, and no subjects getting worse. Mean improvement in the CGI rating of 1.61 corresponded to a mild-to-moderate overall improvement. Likewise, the VAS showed significant improvement in 12 of the 19 subjects in parent-defined behaviours, while 6 remained unchanged and 1 became worse. The parent-defined behaviours included attention deficit, perseveration, anxiety, self-injurious behaviour, abnormal vocalizations, mood swings, social avoidance and repetitive behaviour. No significant differences were found on any of the outcome measures between the high dose and low dose groups, and this raises the possibility of a placebo effect. Furthermore, participants taking additional medications did not differ on any of the outcome measures compared to those taking only minocycline.

**Table 3:** Baseline Scores and Group Change in Behaviour after Treatment with Minocycline for 8 weeks

	Baseline Group		8 Week Group		Group Mean		**p**^**1**^
	
Measure	Mean	+/- SD	Mean	+/- SD	Change	+/- SD	
**VAS**	19.32	7.67	34.40	18.48	15.58	20.25	.003

**CGI**	4.67	0.49	3.06	0.64	-1.61	0.78	< .001

**ABC-C**^2^							

Irritability	22.11	7.23	7.74	4.82	-14.37	7.84	< .001

Lethargy	15.37	10.44	11.47	6.28	-3.89	8.30	.065

Stereotypy	7.95	5.34	3.79	3.07	-4.16	3.34	< .001

Hyperactivity	17.42	10.43	11.11	7.06	-6.32	7.67	.002

Inappropriate speech	7.42	4.00	4.79	2.68	-2.63	3.13	.002

Total	70.26	26.03	38.90	17.78	-31.37	23.21	< .001

1 Significance with Bonferroni correction applied.

2 Negative values indicate a positive change.

At the end of 8 weeks of treatment, subjects were offered ongoing treatment with follow up through an extension study for 1 year. At 8 weeks, eighteen families independently reported an improvement in some level of functioning and elected to continue the one year extension. One participant did not continue beyond 4 weeks of treatment because of the limiting gastrointestinal side effects, and the other chose to leave the trial after 8 weeks because the parents did not feel there had been any significant benefit to treatment with the minocycline and did not want to continue with the follow up visits.

## Discussion

The results of this pilot open-label trial suggest that minocycline has positive effects on behavioural symptoms in individuals with FXS. Significance was reached for the primary endpoint, change in the ABC-C Irritability Subscale, as well as five out of six secondary outcome measures. If, as hypothesized for this study, minocycline targets an underlying molecular defect in FXS, then it would be expected to improve multiple areas of dysfunction and this is suggested by our data. Lethargy was the only area that did not show any statistically significant changes.

Positive responses were distributed across the age range of the study cohort, suggesting that both adolescents and adults with FXS can benefit from minocyline treatment. Beneficial effects of minocycline in this small cohort did not seem to relate strongly to dose, although it is possible that such relationships would emerge in a larger treatment cohort. Also, since this trial of minocycline was adjunctive or add-on therapy, half of the subjects were on other medications to manage their behavioural dysfunction and even though doses were not changed, it is possible that interactions between minocycline and these medications could have contributed to the minocycline response. Although there appeared to be statistically significant pre-post treatment differences on measures of irritability and lethargy as a function of medication, these differences were not significant when the Bonferroni correction methods were applied and a more conservative significance value (i.e., p = 0.01) was applied. Furthermore, the size of the cohort was too small to draw any conclusions regarding the interaction with different classes of psychoactive medications that the participants were taking.

All subjects were in a consistent educational or work program throughout the 8 weeks in the trial. Although environmental factors could have contributed to the minocycline response, and minor environmental changes would be difficult to control, there were no substantial changes in programming, family environment or living setting during the treatment period for any subject.

Dizziness and diarrhea were the most commonly reported side effects during this trial. Symptoms of sleepiness, headache, fatigue, nausea and pruritus were other complaints occurring infrequently during treatment in this cohort that may represent side effects of minocycline in FXS. Of particular note however, were the asymptomatic seroconversions to a positive ANA in 2 trial subjects. Neither of these subjects had any rheumatologic or constitutional symptoms but they were not allowed to continue the trial beyond 8 weeks. At least 15 cases of minocycline-induced lupus-like symptoms have been reported in the literature [36]. A retrospective cohort study of patients with development of rheumatologic symptoms while receiving minocycline showed that a substantial proportion of children with minocycline-induced autoimmunity can develop chronic symptoms with the potential for significant morbidity [37]. Physicians who prescribe minocycline should be aware of its propensity for inducing potentially serious autoimmune phenomena. Other potential risks, such as the prolonged use of an antibiotic, will have to be weighed against the possible benefits of using minocycline to treat patients with FXS. However, minocycline is already indicated for long-term use to treat acne. Adolescents with acne are frequently treated with minocycline for periods of months or years, and this drug has proven to be very well tolerated [38,39].

There are a number of limitations to this study, specifically the small size of the cohort treated and the open-label design, which allows for potential placebo effect and rater bias. Use of a second rater and inter-rater reliability comparisons would have helped improve the validity of these measures. Furthermore, the usage of the ABC and CGI assessments are also limited in this disorder, partly because of the difficulty defining the underlying basis to the externalizing behaviours and partly because assessment of these individuals is difficult in itself because of the dynamic behavioural issues. However, there have been relatively few clinical drug trials in patients with fragile X, and these tools appear to be the best available outcome measures at this time.

The concept motivating this study was to extend to a human population the findings that minocycline can normalize the phenotype seen in the mouse model for FXS. In that regard the trial was aimed at targeting an underlying molecular defect in FXS as a mode of treating the behavioural phenotype in humans. Theoretically, minocycline should act by inhibiting excessive MMP activity, which is known to be involved in normal synaptic structure and function [40], and is excessively translated due to the lack of normal translational repression by FMRP. Furthermore, minocycline is not thought to possess any significant intrinsic anxiolytic or psychotropic properties, though two recent studies in schizophrenic subjects suggest that it may augment the effect of antipsychotics [41,42]. Yet minocycline treatment resulted in significant improvements across a broad range of behaviours seen in FXS, with only one trial participant receiving concomitant antipsychotic medication. While these results could be due to a placebo effect, the findings suggest that minocycline may be targeting an underlying molecular defect in this disorder and the results of this trial are consistent with the positive effects of minocycline in the mouse model of FXS.

## Conclusions

The results of this study are consistent with the concept that modulation of excessive MMP production would be helpful in FXS, and suggest that a placebo-controlled trial of minocycline in FXS should be done as a next step to confirm effectiveness, before generally recommending minocycline treatment to individuals with FXS. While adverse events would not be expected to be a limiting problem in such a controlled trial, screening of ANA titres with follow-up during the treatment period would be recommended to avoid potential cases of minocycline induced autoimmunity. This pilot study serves as an example to demonstrate the translation of information from basic science and animal model research to the clinical treatment of a neurodevelopmental disorder.

## Competing interests

The authors declare that they have no competing interests.

## Authors' contributions

CP participated in the design of the study, submitted all trial applications, conducted the medical assessments of the subjects, and drafted the manuscript. LT participated in trial coordination and assisted with medical assessments of the trial subjects. AF conducted the behavioural and cognitive assessments of the subjects, and performed the statistical analysis on the data. EBK participated in the design of the study. MT conceived of the study, and participated in its design. IME conducted the preclinical studies on the *fmr1 *knockout mouse. DWE conducted the preclinical studies on the *fmr1 *knockout mouse. All authors have read and approved the final manuscript.

## Pre-publication history

The pre-publication history for this paper can be accessed here:

http://www.biomedcentral.com/1471-2377/10/91/prepub
